# Comment on Article “Postoperative Serum Hyperamylasemia Adds Sequential Value to the Fistula Risk Score in Predicting Pancreatic Fistula After Pancreatoduodenectomy”

**DOI:** 10.1097/AS9.0000000000000391

**Published:** 2024-02-09

**Authors:** Ziqiang Du, Rui Du, Yanfei Yang

**Affiliations:** * From the Department of General Surgery, The First Affiliated Hospital of Dalian Medical University, Dalian, China.

We have recently been very interested and discussed in depth an article by Bannone et al.^1^ The author evaluates whether postoperative serum hyperamylasemia, with drain fluid amylase and C-reactive protein, improves the Fistula Risk Score (FRS) accuracy in assessing the risk of a postoperative pancreatic fistula (POPF).^1^ We appreciate the author what have done for the research, but there are still some deficiencies that we need to discuss together.

First of all, according to relevant reports, the ratio of drainage fluid to serum amylase concentration (area under the curve: 0.77, sensitivity: 77.7%, specificity: 73.3%, and accuracy: 73.6%) is an independent risk factor for POPF, and this risk factor deserves to be included in the POPF prediction model for modeling, so as to improve the comprehensiveness of the FRS.^2^ Some relevant studies have reported that inflammatory burden index stands out among related biomarkers of inflammation for tumor prognosis. If inflammatory burden index is included in the exploratory study of FRS rather than the simple study of C-reactive protein, the scientific, comprehensive, and rigorous nature of the FRS constructed will be greatly improved.^3^ At the same time, some relevant studies have reported that the prediction of postoperative acute pancreatitis may have a better prediction effect. If the author combined the above 2 viewpoints with the prediction of postoperative serum hyperamylasemia and postoperative acute pancreatitis to explore FRS, it will be more meaningful.^4^ Finally, we would like to thank the authors again for their contribution. This study provides a more comprehensive and logically rigorous guide for the exploration of FRS, which is of great significance.
